# Biomechanical Investigation of the Stomach Following Different Bariatric Surgery Approaches

**DOI:** 10.3390/bioengineering7040159

**Published:** 2020-12-09

**Authors:** Ilaria Toniolo, Chiara Giulia Fontanella, Mirto Foletto, Emanuele Luigi Carniel

**Affiliations:** 1 Department of Industrial Engineering, University of Padova, Via Venezia 1, 35131 Padova, Italy; ilaria.toniolo.1@phd.unipd.it (I.T.); emanueleluigi.carniel@unipd.it (E.L.C.); 2 Centre for Mechanics of Biological Materials, University of Padova, Via F. Marzolo 9, 35131 Padova, Italy; mirto.foletto@unipd.it; 3 IFSO Bariatric Center of Excellence, Padova University Hospital, Via Ospedale Civile, 35121 Padova, Italy

**Keywords:** stomach biomechanics, bariatric surgery, computational model, anisotropic visco-hyperelastic model, finite element analysis

## Abstract

Background: The stomach is a hollow organ of the gastrointestinal tract, on which bariatric surgery (BS) is performed for the treatment of obesity. Even though BS is the most effective treatment for severe obesity, drawbacks and complications are still present because the intervention design is largely based on the surgeon’s expertise and intraoperative decisions. Bioengineering methods can be exploited to develop computational tools for more rational presurgical design and planning of the intervention. Methods: A computational mechanical model of the stomach was developed, considering the actual complexity of the biological structure, as the nonhomogeneous and multilayered configuration of the gastric wall. Mechanical behavior was characterized by means of an anisotropic visco-hyperelastic constitutive formulation of fiber-reinforced conformation, nonlinear elastic response, and time-dependent behavior, which assume the typical features of gastric wall mechanics. Model applications allowed for an analysis of the influence of BS techniques on stomach mechanical functionality through different computational analyses. Results: Computational results showed that laparoscopic sleeve gastrectomy and endoscopic sleeve gastroplasty drastically alter stomach capacity and stiffness, while laparoscopic adjustable gastric banding modestly affects stomach stiffness and capacity. Moreover, the mean elongation strain values, which are correlated to the mechanical stimulation of gastric receptors, were elevated in laparoscopic adjustable gastric banding compared to other procedures. Conclusions: The investigation of stomach mechanical response through computational models provides information on different topics such as stomach capacity and stiffness and the mechanical stimulation of gastric receptors, which interact with the brain to control satiety. These data can provide reliable support to surgeons in the presurgical decision-making process.

## 1. Introduction

Bariatric surgery (BS) is considered the most effective treatment for severe obesity that aims to achieve weight loss and resolve metabolic comorbidities [1,2]. BS mainly focuses on the stomach and the intestines, which are involved in food digestion and nutrient adsorption. The gut also controls satiety and satiation by means of the so-called gut–brain axis [3]. In fact, the gastric wall is populated by mechano-chemical receptors, the actions of which were recorded by several electro-physiological experimental studies [4,5,6]. Although the receptors in mucosa can identify the physical and chemical natures of luminal content, muscle receptors are mainly influenced by changes in deformation of the wall. In the muscular layer, at least two morphological types of vagal afferent endings occur: intraganglionic laminar endings (IGLEs) and intramuscular arrays (IMAs) [6,7]. The latter are highly concentrated in the upper stomach [8,9]. Food ingestion determines the mechanical stimulation of the gut wall and the production of specific chemical and biochemical compounds. Receptors transduce these stimuli into hormonal release and neural responses. The brain stores and encodes all these various nature responses and elicits the feeling of satiety [10]. The study of satiety is very complex because it depends on the intensity and type of mechanical and chemical stimuli, but also on adiposity signals, personal habits, and social and emotional situations [11].

Many different bariatric interventions have been proposed and designed to reduce stomach capacity and increase stomach stiffness or to induce satiety and/or malabsorption phenomena. The American Society for Metabolic and Bariatric Surgery estimated that in 2018, the total number of bariatric procedures amounted to 252,000, with an increase of 10.8% with respect to 2017: 61.4% were laparoscopic sleeve gastrectomy (LSG) procedures, 17% were roux-en-Y gastric bypass (RYGB) procedures, 1.1% were adjustable gastric banding (AGB) procedures, 0.8% were biliopancreatic diversion (BPD-DS) procedures, 15.4% were revision operations, 2% were balloons, and 2.3% were other procedures. In general, the number of gastric bypasses has decreased since 2011, but an inverse trend was recorded from 2017 to 2018, with an increase of 5.8%, whereas AGB operations are still in decline [12]. Aiming for a less invasive approach, endoscopic BS techniques have already spread worldwide: in recent years, various types of endoluminal interventions, such as endoscopic sleeve gastroplasty (ESG), and other devices have also been developed, with varying levels of surgical invasiveness, to safely treat high-risk patients [13].

**Figure 1:**
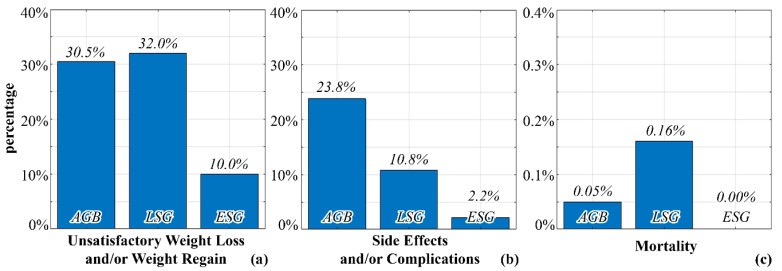
Identification of the weaknesses of traditional and current bariatric surgery (BS) techniques, with particular regard to unsatisfactory weight loss and/or weight regain (**a**), side effects and/or postsurgical complications (**b**), and mortality (**c**) [15,16,17,18,19,20,21,22,23,24].

Currently, there are no specific guidelines for the selection of the optimal procedure. Usually, clinical evidence, the surgeon’s experience, and the patient’s medical conditions inform the choice of bariatric procedure. Surgical procedures are characterized by different postsurgical complications and drawbacks: hernias and ulcers can occur after RYGB, whereas AGB must deal with complications related to band position, migration, and erosion. The most controversial issue for LSG is gastroesophageal reflux disease due to both the increase of esophagogastric junction angle and the strong reduction of the stomach [14]. In general, unsatisfactory weight loss or weight gain is a possible postsurgical complication, and altered food approaches affect up to 25% of bariatric patients [15,16,17,18,19,20,21,22,23,24] (Figure 1), leading to the need for improvements in BS.

A computational approach to BS allows us to analyze the influence of bariatric intervention on stomach capacity and stiffness, further providing information that experimental methods are not able to supply with a noninvasive method, such as strain and stress field distributions within biological tissues. Strain describes the shape modification that the biological tissue locally experiences, whereas stress specifies the mechanical actions that the tissue locally senses. The knowledge of such mechanical stimuli is crucial in the field of surgery because they regulate many different mechano-biological effects, such as tissue damage or failure, tissue adaptation, and mechano-transduction phenomena [25,26]. The mechanical stimulation of the stomach wall, as strain distribution, cannot be collected in vivo because of the invasive equipment and the difficulty in measuring large elongation strain in soft biological tissues. On the other hand, several electrophysiological studies investigating IGLEs and IMAs have been performed on animal models [27], reporting on the influence of wall distention on satiety. In this sense, the evaluation of mechanical stimulation of the gastric wall is fundamental for the comprehension of the effects of bariatric intervention on the mechanisms of satiety and satiation [28]. It follows that the methods of computational biomechanics allow an engineering contribution to BS intervention design that is aimed at providing valid support to surgeons in the presurgical decision-making process and success rate prediction, thus raising the success rate of surgical procedures and devices [29,30].

**Figure 2:**
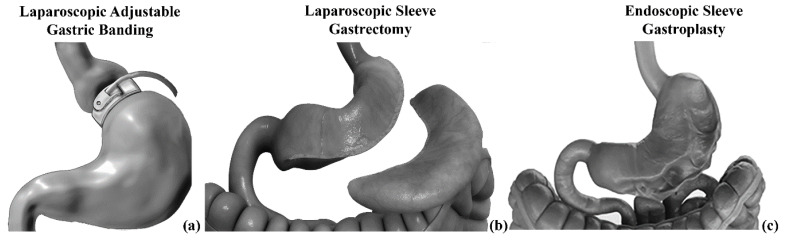
The investigated BS techniques: laparoscopic adjustable gastric banding (**a**), laparoscopic sleeve gastrectomy (**b**), and endoscopic sleeve gastroplasty (**c**).

In this paper, computational modeling of stomach mechanics is described and developed to analyze the effects of BS. Mechanical functionality was investigated by employing the classical engineering approach, which is based on the definition of physical models of biological structure by coupling experimental and computational activities. Experimental tests are necessary for model definition, identification, and validation. Subsequently, computational methods allow for the expansion of experimental results to a wider scenario, considering the configurations of biological structure in different surgical procedures. In this work, only surgical techniques that mainly involve the stomach and its shape modification are considered, such as AGB, LSG, and ESG (Figure 2). RYGB was not taken into consideration because, even though it modifies the stomach by leaving a small punch, the weight-loss mechanism is mainly achieved by altering the adsorbing mechanism and not by modifying stomach mechanical functionality.

## 2. Materials and Methods

### 2.1. Computational Mechanical Model Definition

The computational mechanical model of the stomach characterizes both its geometrical conformation and the mechanical behavior of building tissues. Model development requires coupled experimental and computational activities [31,32].

The stomach model was developed based on a computational modeling approach [33,34]. Tomographic techniques, with particular regard to magnetic resonance imaging (MRI), along with image analyses and segmentation procedures, have led to the computer-aided design virtual model, whose discretization, performed by preprocessing computer-aided engineering software, provides the geometrical definition of the computational model [33,34]. The model is characterized by the following average dimensions, evaluated by considering different stomach measurements [35,36]: 19 cm height, 15 cm average width, 38 cm long greater curvature, 8 cm long lesser curvature, 2 mm thickness in the fundus region, 3 mm thickness in the corpus region, and 3.5 mm thickness in the antrum region. Finite element discretization was performed using 10-node tetrahedral elements, with an average element size of 0.5 mm. The assumed configuration led contemporarily to a computationally light model and to a suitable number of nodes along the wall thickness, which is mandatory for a reliable evaluation of stress and strain fields. The final model consisted of about 370,000 elements and 74,000 nodes.

Data from histological investigations and mechanical tests on tissue samples allowed for the characterization of the mechanical response of stomach tissues using constitutive formulations. The performed constitutive analysis envisaged the typical features of gastrointestinal tissues, such as the multilayered and fiber-reinforced conformation, the nonlinear elastic response, and the time-dependent behavior, leading to an anisotropic visco-hyperelastic formulation [32,34]. The second Piola–Kirchhoff stress tensor **S** was computed according to principles of thermodynamics, as follows:

(1) $$S\left(C,q^{i}\right)=2\partial W^{0}\left(C\right)/\partial C-\sum_{i}q^{i}$$

where C is the right Cauchy–Green strain tensor and $W^{0}$ is the strain energy function that specifies the instantaneous hyperelastic mechanical response of the layer, whereas $q^{i}$ are viscous internal variables that specify relaxation phenomena. The strain energy function was then defined by the following formulations:

(2) $$W^{0}\left(C\right)=W^{0m}\left(C\right)+W^{0f}\left(C,a_{0},b_{0}\right)$$

(3) $$W^{0m}\left(C\right)=-p\left({I_{3}}^{1/2}-1\right)+\left[C_{1}/\alpha_{1}\right]\left\{\exp\left[\alpha_{1}\left(I_{1}-3\right)\right]-1\right\}$$

(4) $$W^{0f}\left(C,a_{0},b_{0}\right)=\frac{C_{4}}{{\alpha_{4}}^{2}}\left\{\exp\left[\alpha_{4}\left(I_{4}-1\right)\right]-\alpha_{4}\left(I_{4}-1\right)-1\right\}+\frac{C_{6}}{{\alpha_{6}}^{2}}\left\{\exp\left[\alpha_{6}\left(I_{6}-1\right)\right]-\alpha_{6}\left(I_{6}-1\right)-1\right\}$$

where $a_{0},b_{0}$ are unit vectors that specify layer preferential orientations in the unstrained configuration, $I_{1}$ and $I_{3}$ are the first and the third invariants of the right Cauchy–Green strain tensor, while $I_{4}$ and $I_{6}$ are structural invariants that specify the square of tissue stretch along directions $a_{0}$ and $b_{0}$, respectively. The term *p* is a Lagrange multiplier that ensures the incompressibility constraint. Constitutive parameter $C_{1}$ specifies the tissue’s initial shear stiffness, while parameter $\alpha_{1}$ regulates the nonlinearity of the shear response. Parameters $C_{4}$ and $C_{6}$ are constants that define the fibers’ initial stiffness, while $\alpha_{4}$ and $\alpha_{6}$ depend on fibers stiffening with stretch.

The evolution law for viscous variables $q^{i}$ was defined by means of differential equations depending on specific viscous parameters, such as relative elastic stiffness $\gamma^{i}$ and relaxation time $\tau^{i}$:

(5) $${\overset{\cdot }{q}}^{i}+\frac{1}{\tau^{i}}q^{i}=2\frac{\gamma^{i}}{\tau^{i}}\frac{\partial W^{0}}{\partial C}$$

The next step of the constitutive characterization pertains to the identification of constitutive parameters, which are usually based on the inverse analysis of experimental tests [33,37,38]. The identification of visco-hyperelastic parameters requires data from in vitro experimentations on tissue and structure specimens because in vivo experimentations cannot be easily performed. Results from previous experimental tests were adopted, as fully reported by Fontanella et al. [34]. Preliminarily, the analysis of tensile tests on tissue specimens by Zhao et al. [39] led to sets of constitutive parameters for connective stratum and muscularis externa of fundus, corpus, and antrum regions. Subsequently, the computational model of the stomach was exploited to simulate mechanical tests at the structure level, such as inflation tests [29,33,34,40]. Constitutive parameters were updated to the agreement between model results and median experimental data, providing a reliability assessment of the developed computational framework.

### 2.2. Computational Mechanical Model Exploitation

**Figure 3:**
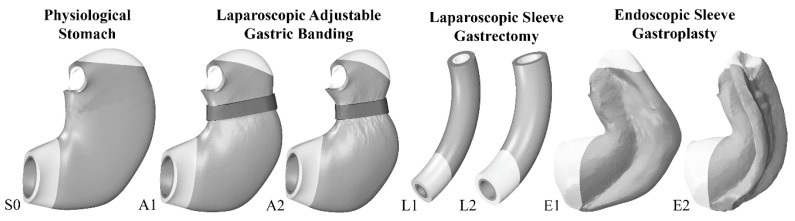
Computational mechanical models of the stomach before (S0) and after BS: laparoscopic adjustable gastric banding (A1: 25% band pretensioning; A2: 40% band pretensioning), laparoscopic sleeve gastrectomy (L1: 32 Fr bougie size; L2: 40 Fr bougie size), and endoscopic sleeve gastroplasty (E1: two-point stitch configuration; E2: three-point stitch configuration). Model development accounts for the principal regions of the stomach, such as fundus (white), corpus (grey), and antrum (white).

The biomechanical model of the stomach allowed for the in-silico investigation of stomach mechanical functionality, considering the most common bariatric procedures, such as LAGB, LSG, and ESG. Consequently, the computational model of the presurgical stomach (S0) was modified, and specific analyses were performed to investigate the different BS procedures (Figure 3). To mimic the LAGB technique, a computational model of the silicone band was implemented, and it was wrapped around the stomach model along a greater-to-lesser curvature direction. A hyperelastic formulation was assumed to characterize the mechanical behavior of the silicone band, and a friction contact condition (0.1 friction coefficient) was permitted to simulate the actual interaction phenomena occurring between the band and the stomach. The simulations accounted for different band pretensioning conditions (A1, A2). Concerning LSG surgery, a tubular model of the stomach was developed. According to the surgical procedure, the model included the corpus and antrum regions of the stomach only. Intraluminal diameters of 32 and 40 Fr were analyzed (L1, L2). Finally, computational techniques allowed for the simulation of the endoscopic suture that is performed during ESG, along with the entire stomach structure, in a proximal-to-distal direction. Wire elements were used to connect the anchoring points of the sutures, bringing the involved surfaces up to contact. Two variants of ESG were simulated: fixation between the front and rear walls of the stomach (two-point stitch configuration; E1); stitching of the front wall, the rear wall, and the great curvature (three-point stitch configuration; E2). Wall thickness and the material properties of the tissue layers were preserved for each bariatric procedure as in the presurgical model.

**Figure 4:**
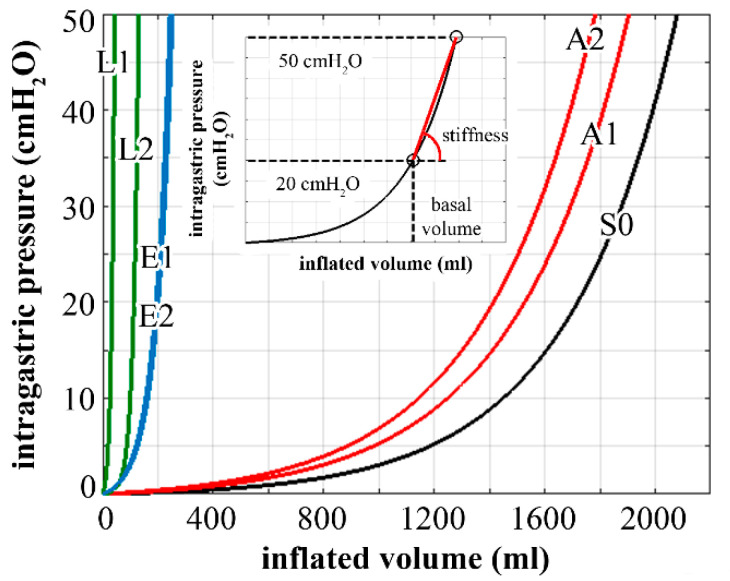
Computational analysis of stomach inflation: pressure–volume behavior of physiological stomach (S0), and postsurgical stomach after laparoscopic adjustable gastric banding (A1, A2), laparoscopic sleeve gastrectomy (L1, L2), and endoscopic sleeve gastroplasty (E1, E2). The subplot reports the procedure for the calculation of stomach basal volume and stiffness.

For each configuration, stomach biomechanical functionality was evaluated by performing an inflation process of up to 50 cm H_2_O intragastric pressure. Gastroesophageal and duodenal junctions were kept fixed during the inflation process. The computational analyses led to pressure–volume curves (Figure 4), the processing of which allowed for the identification of stomach basal volume, such as the stomach volume at 20 cm H_2_O intragastric pressure, and stomach stiffness, such as the average slope of the pressure–volume curve in the 20–50 cm H_2_O pressure range (Figure 4). All simulations were performed using the general-purpose finite element code Abaqus Standard 2018 (Dassault Systèmes, Simulia Corp., Providence, RI, USA).

## 3. Results

A computational mechanical model of the stomach was used to investigate gut functionality in both physiological (S0) and postsurgical conformations (Figure 3). In detail, different bariatric techniques were analyzed, such as LAGB (A1: 25% band pretensioning; A2: 40% band pretensioning), LSG (L1: 32 Fr bougie size; L2: 40 Fr bougie size), and ESG (E1: two-point stitch configuration; E2: three-point stitch configuration).

**Figure 5:**
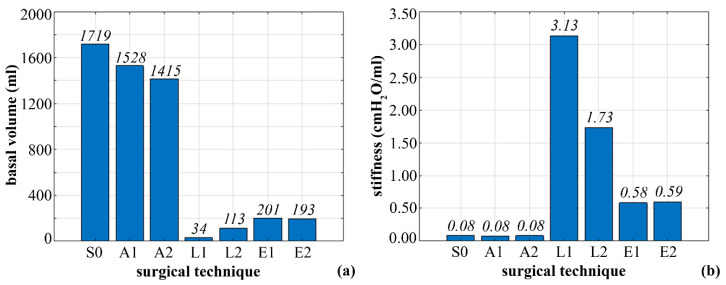
Computational results from simulations of stomach inflation (physiological stomach S0; postsurgical stomach with laparoscopic adjustable gastric banding A1 and A2, laparoscopic sleeve gastrectomy L1 and L2, and endoscopic sleeve gastroplasty E1 and E2): comparison of stomach basal volumes (**a**) and stiffness (**b**).

The resulting basal volumes were 1719, 1528, 1415, 34, 113, 201, and 193 mL, and the measured stiffness amounted to 0.08, 0.08, 0.08, 3.13, 1.73, 0.58, and 0.59 cm H_2_O/mL for S0, A1, A2, L1, L2, E1, and E2 configurations, respectively (Figure 5).

**Figure 6:**
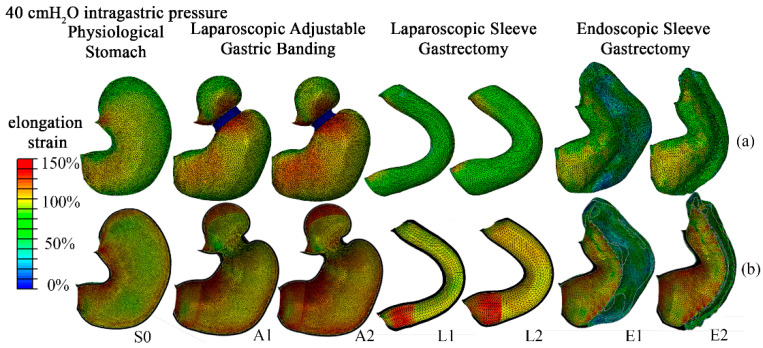
Contours of stomach distension as tissue elongation strain at 40 cm H_2_O intragastric pressure: comparison of results for physiological stomach (S0), laparoscopic adjustable gastric banding (A1, A2), laparoscopic sleeve gastrectomy (L1, L2), and endoscopic sleeve gastroplasty (E1, E2) for the outer (**a**) and inner layers (**b**).

The computational analyses provided information about the distribution of stress and strain fields, which are responsible for the mechanical stimulation of gastric receptors. Implementation of postsurgical modifications of stomach conformation permitted us to evaluate the influence of BS on the mechanical stimulation of gastric receptors. The contours of the elongation strain are reported in Figure 6.

**Figure 7:**
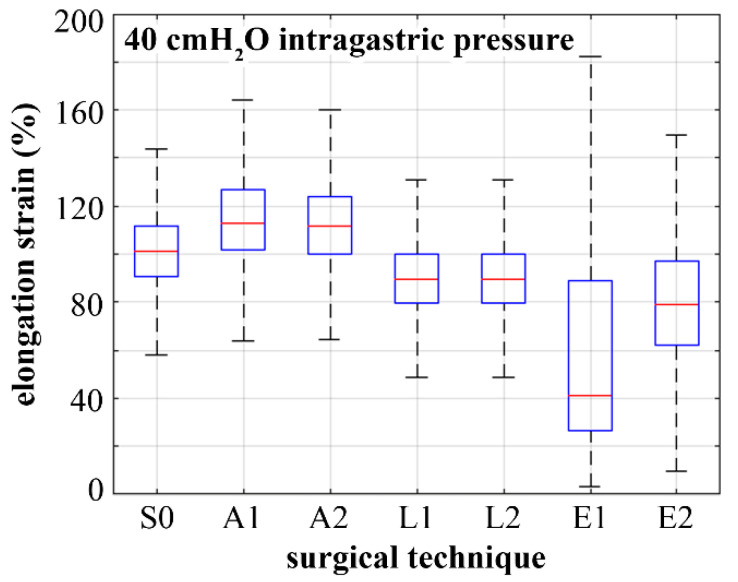
Box plots representing the distribution of stomach distension as tissue elongation strain within the overall stomach wall: comparison of results for the physiological stomach (S0), laparoscopic adjustable gastric banding (A1, A2), laparoscopic sleeve gastrectomy (L1, L2), and endoscopic sleeve gastroplasty (E1, E2).

With the aim of evaluating the influence of BS on the comprehensive mechanical stimulation of the gastric wall, tissue elongation strain was evaluated by considering all the points of the stomach models. Subsequently, analyses of statistical distributions were performed. In Figure 7, the 95% confidence intervals were reported for both pre- and postsurgical conditions. The mean elongation strain values were 100%, 114%, 112%, 91%, 91%, 56%, and 79% for S0, A1, A2, L1, L2, E1, and E2 configurations, respectively. The layer that recorded the higher values of elongation strain was the inner one, such as the submucosa–mucosa layer, for all the configurations, especially in the fundus region, if this region was present. In the L1, L2, E1, and E2 configurations, the inner layer of the antrum region presented higher elongation strain values than the corpus layer. For endoscopic bariatric procedures, the external layer recorded greater values of elongation strain than the laparoscopic layer, especially in the antrum region. In A1 and A2 configurations, high elongation strain values took place in the external layer, such as the muscularis stratum, in the corpus region where the bandings were positioned (about 135% elongation strain), and in the antrum region near the duodenum (about 95% elongation strain).

## 4. Discussion

Obesity is an epidemic disease associated with multiple comorbidities, the prevalence of which, in developed countries, is increasing. Behavioral and pharmacological treatments are modestly effective, whereas BS remains the most effective approach. Nonetheless, unsatisfactory weight loss or weight regain, side effects, and postsurgical complications may affect a significant percentage of patients. In some cases, reoperative surgery, following the failure of primary BS, is needed [41,42,43]. With particular regard to restrictive procedures such as LSG and LAGB, the current global incidence of reoperations is estimated to be as high as 50%. Procedures of reoperative BS have a high level of complexity and are technically demanding [43]. Since bariatric procedures are mainly based on clinical experience, a more rational approach should be advocated. The causes of failure can be analyzed by considering the biomechanical aspects. Bariatric operations aim to reduce food and calorie intake by modifying stomach capacity and stiffness and controlling meal-induced satiety. Satiety occurs through complex interactions between the gut and the brain via neural and hormonal signals. Food intake leads to mechanical and chemical stimulation of the stomach wall where gastric receptors are located. Receptors turn stimuli transduction into neural and hormonal signals that are all conveyed to the brain, which, in turn, elicits satiety according to stimulation intensity. The results proposed in this paper highlight the altered mechanical functionality of the stomach after the intervention, giving additional information to clinicians.

The methods of biomechanics may provide useful tools for the rational comprehension of BS [29,40]. To emphasize the potential of the computational approach, a stomach biomechanical model was developed and utilized to analyze postsurgical configurations and to compare different bariatric techniques. In detail, the computational approach provides quantitative information about stomach mechanical functionality in pre- and postsurgical conformations. The versatility of the in-silico approach allows for the exploration of the influence of surgical parameters such as band inflation and pretensioning for LAGB, bougie size for LSG, and stitch configuration for ESG (Figure 3). Computational results point out the actual influence of surgical parameters on stomach mechanical functionality concerning stomach capacity and stiffness (Figure 4 and Figure 5) but also mechanical stimulation of gastric receptors (Figure 6 and Figure 7). The comparison of results from the computational simulation of different bariatric interventions quantitatively reveals the mechanisms of action of the different techniques: LSG and ESG drastically alter stomach capacity and stiffness, but they also strongly reduce the mechanical stimulation of gastric receptors concerning mean values; however, in ESG, the data dispersion is elevated, with peaks of about 180% of elongation strain; LAGB modestly affects stomach stiffness and capacity, but it boosts the stimulation of gastric receptors.

By comparing model results with data from clinical experience, stomach model capacities such as basal volume in the physiological condition (S0) and after laparoscopic sleeve gastrectomy (L1 and L2) or laparoscopic gastric banding (A1 and A2) agree with the clinical values reported in the literature [44]; however, it is quite difficult to make a good comparison because clinical activities aim to give average values of volumetric capacity, which strongly depends on intragastric pressure and is affected by intersample variability. On the other hand, the computational approach makes it possible to evaluate stomach capacity depending on intragastric pressure and surgical procedure. Concerning gastric wall distension, reports in the scientific literature have investigated the influence of surgical procedures on mechanisms of satiety [10,11,45]. In vivo measurement of such elongation strains is quite complex. Only a few studies have reported quantitative values [46,47,48] that have a magnitude in the range of results from our proposed computational investigations; however, the computational approach allows for a fully quantitative and detailed evaluation of such mechanical inputs. Further activities are under development at the University of Padova, which aim to correlate food intake, mechanical stimulation of gastric receptors, brain activation, and feeling of satiety. The analysis envisages coupled computational analyses, stomach MRI measurements, and brain fMRI investigations [28,49,50].

The main limitations of the proposed work include the use of average geometries and material properties for the stomach models in different BS procedures. The reported modeling approach accounts for structural, mechanical aspects only. Bolus action on the stomach wall is simulated utilizing volumetric inflation of intraluminal space. A more refined framework should also investigate the actual interaction phenomena between bolus and stomach tissues using a fluid–structure interaction (FSI) approach because of the (mostly) fluid nature of the bolus. On the other hand, the complexity of FSI models greatly increases the computational effort of the models, and a purely solid mechanics approach entails the optimal compromise between model reliability, feasibility, and manageability. Moreover, aiming at providing a patient-specific mechanical characterization of stomach tissues by overcoming the limitation of an average computational model, constitutive parameter upgrades and tuning can rest on data from patient biomedical images or ultrasound measurements (e.g., electrographic techniques provide relationships between such data and tissues’ initial elastic properties) [51,52]. The collection of data from patients’ biomedical images and experimentation on human samples is ongoing at the University of Padova.

## 5. Conclusions

The computational model allows the in-silico investigation of stomach mechanical functionality, permitting us to compare BS procedures by showing mechanical quantities that, in vivo, can only be collected with invasive tools. In literature, a rational approach that is able to quantitatively compare BS techniques is still lacking. In fact, the principal considerations of surgical effectiveness are related to postsurgical outcomes, like weight loss and the occurrence of side effects. The reported results are a step towards a more rational approach to BS, highlighting that computational modeling techniques can be a valid clinical tool for the presurgical planning process, ensuring better tailoring and prediction of efficacy. The coupling of bioengineering methods with surgical practice can modify the way of proceeding in the treatment of obesity, reducing social and health system costs.
